# Selenium and gastrointestinal cancers risk

**DOI:** 10.1186/1897-4287-10-S4-A3

**Published:** 2012-12-10

**Authors:** Marcin Lener, Anna Jakubowska, Anna Wiechowska-Kozłowska, Józef Kładny, Magdalena Muszyńska, Grzegorz Sukiennicki, Jan Lubiński

**Affiliations:** 1 Department of Genetics and Pathology, Pomeranian Medical University, Szczecin, Poland; 2 Laboratory of Endoscopy, Division of Health Care Ministry of Internal Affairs and Administration, Szczecin, Poland; 3 Department of General and Oncological Surgery, Pomeranian Medical University, Szczecin, Poland

## 

Research suggests that selenium may influence the behavior of the cancer risk in two ways. As an antioxidant, selenium helps to protect the body against free radicals. Selenium may also prevent or slow tumor growth, as some breakdown products of selenium can inhibit tumor growth by enhancing immune cell activity and inhibition of tumor blood vessel development.

## Aim

The aim of this study was to determine the level of selenium in blood serum as a potential marker of risk for cancers of the colon, stomach or pancreas.

## Material and methods

The research material was a total of 110 samples of blood serum from people with cancer, diagnosed and confirmed in one of the organs: colon (67 cases), pancreas (30 cases) or stomach (13 cases) and 110 samples of blood serum derived from healthy individuals representing paired control group. The criteria adopted for pairing included: gender, year of birth (+/- 3 years), history of the occurrence of cancers in the family among first degree relatives and smoking status expressed in pack-years.

Selenium concentration in blood serum was determined using inductively coupled plasma mass spectrometry (ICP-MS) – Perkin Elmer. Validation: Seronorm^TM^, Nycomed Pharma AS, Oslo, Norway. The measurement accuracy was +/- 5% µg Se/l.

## Results

Association between Se concentration and frequency of cancers in quartiles are presented in table 1. Statistical analyses are summarized in table 2.

**Table 1:** Association between Se plasma concentration and risk of cancers analyzed.

Cancer site	Quartile	**Se concentration range (*****µg/l*****)**	No. of Cases/ Contorls
**Pancreas**	**I**	25,69-50,09	15/0
	**II**	50,72-65,58	9/6
	**III**	66,34-73,30	6/9
	**IV**	74,07-113,89	0/15

**Colorectal and stomach**	**I**	15,92-56,75	35/5
	**II**	57,3-67,91	22/18
	**III**	68,13-78,07	12/28
	**IV**	78,38-155,72	11/29

**Table 2:** Results of statistical analyses of cancer site depending on Se concentration.

Cancer site	Quartiles compared	**Se concentration range (*****µg/l*****)**	Cases/controls in compared groups	Fisher’s Exact Test		
	
				P	OR	CI
**Pancreas**	I vs II	25,69-50,09 vs 50,72-65,58	15/0 vs 9/6	0,017	21,2	1,07-421,11
	I vs III	25,69-50,09 vs 66,34-73,30	15/0 vs 6/9	0,0007	45,3	2,2-899,53
	I vs IV	25,69-50,09 vs 74,07-113,89	15/0 vs 0/15	<0,0001	961	7,9-51,617

**Colorectal and stomach**	I vs II	15,92-56,75 vs 57,3-67,91	(5)*35/5 vs (4)*22/18	0,0026	5,7	1,86-17,66

	I vs III	15,92-56,75 vs 68,13-78,07	(5)*35/5 vs (1)*12/28	<0,0001	16,33	5,14-51,89
	I vs IV	15,92-56,75 vs 78,38-155,72	(5)*35/5 vs (3)*11/29	<0,0001	18,46	5,76-59,25

*stomach cancer cases

## Conclusions

1. There is a very strong correlation between the level of selenium in serum and the risk of gastrointestinal cancers (pancreas, colon, stomach) in the Polish population.

2. Due to the risk of gastrointestinal cancers evaluated most people from the Polish population should increase the level of selenium in serum to approximately 80-100 μg/l.

3. Prospective studies can elucidate:

a) the use of selenium measurements as markers of risk of above cancers,

b) possibility of lowering risk of the cancers of the colon, pancreas and stomach by supplementation of diet with selenium.

4. Assessment of the level of selenium may increase the effectiveness of many screening programs of gastrointestinal cancers, for example, the cost of detecting asymptomatic colorectal cancer by colonoscopy can be reduced dozen times.

